# Characterization of Wnt/β-catenin and BMP/Smad signaling pathways in an *in vitro* model of amyotrophic lateral sclerosis

**DOI:** 10.3389/fncel.2013.00239

**Published:** 2013-12-03

**Authors:** Cristina Pinto, Pilar Cárdenas, Nelson Osses, Juan P. Henríquez

**Affiliations:** ^1^Laboratory of Developmental Neurobiology, Department of Cell Biology, Faculty of Biological Sciences, Center for Advanced Microscopy, Universidad de ConcepciónConcepción, Chile; ^2^Institute of Chemistry, Faculty of Sciences, Pontificia Universidad Católica de ValparaísoValparaiso, Chile

**Keywords:** Wnt, BMP, ALS, motor neurons, beta catenin, smad proteins

## Abstract

Different pathways activated by morphogens of the early embryonic development, such as the Wnt and the Bone Morphogenetic Protein (BMP) ligands, are involved in diverse physiological and pathological conditions of the nervous system, including neurodegeneration. In this work, we have analyzed the endogenous activity of the canonical Wnt/β-catenin and BMP/Smad-dependent pathways in an *in vitro* model of amyotrophic lateral sclerosis (ALS), given by motor neuron-like NSC34 cells stably expressing wild-type or G93A mutated forms of human Cu/Zn superoxide dismutase-1 (SOD1). As ALS-derived motor neurons, NSC34 cells expressing mutated hSOD1 show a decreased proliferation rate, are more susceptible to oxidation-induced cell death and display Golgi fragmentation. In addition, they display an impaired ability to induce the expression of the motor neuronal marker Hb9 and, consistently, to morphologically differentiate into a motor neuronal phenotype. Regarding signaling, our data show that the transcriptional activity associated to the Wnt/β-catenin pathway is decreased, a finding possibly associated to the cytosolic aggregation of β-catenin. In turn, the BMP-dependent phosphorylation of Smad1 and the transcriptional activation of the BMP/Smad pathway is increased in the pathologic model. Together, these findings suggest that Wnt/β-catenin and the BMP-dependent pathways could play relevant roles in the neurodegeneration of motor neurons in the context of ALS.

## INTRODUCTION

Amyotrophic lateral sclerosis (ALS) is a neurodegenerative disease characterized by the selective degeneration and subsequent death of motor neurons, those in the motor cortex and brainstem (superior motor neurons), as well as the ones located in the spinal cord (inferior motor neurons; Goodall and Morrison, 2006; Pasinelli and Brown, 2006). Even though most ALS cases are sporadic, a 5–10% of them have a genetic linkage, as they are associated to point mutations of several proteins, such as Cu/Zn superoxide dismutase-1 (SOD1), TAR DNA-binding protein 43 (TDP43), Fused in Sarcoma (FUS), senataxin (SETX), amongst others (Bruijn et al., 2004; Schymick et al., 2007; Robberecht and Philips, 2013). Remarkably, a 20% of familial ALS cases have been linked to point mutations of SOD1 (Rosen, 1993). Indeed, the G93A mutation of SOD1 has been widely used to generate model systems of ALS, either animals or *in vitro*, as they mimic the main clinical, pathological, and cellular features of the disease (Gurney et al., 1994; Pasinelli et al., 1998; Arciello et al., 2011). The mechanisms by which these mutations cause neuronal death have not been fully described; however, it is believed that rather than changes in protein activity, conformational changes due to these mutations lead to the formation of toxic intracellular protein aggregates (Bruijn et al., 1998; Durand et al., 2006; Bendotti et al., 2012). In spite of the fact that clinical manifestations of ALS occur in the adult life, several research lines indicate that alterations of the motor neuronal network begin at early development of the nervous system, marking the onset of the disease (Bendotti et al., 2012; van Zundert et al., 2012; Robberecht and Philips, 2013). However, the molecular players involved in these early phenotypes have not been fully described.

Cumulative evidence in the last few years reveals that signaling pathways triggered by morphogens of the early embryonic development, such as the Wnt and Bone Morphogenetic Protein (BMP) families, play key roles on different features of the nervous system (Henriquez et al., 2011; Benavente et al., 2012; Henriquez and Salinas, 2012; Salinas, 2012). Wnt ligands signal through their cognate Frizzled (Fz) receptors to activate several different pathways. Upon activation of the “canonical” Wnt pathway, the key intracellular effector β-catenin escapes proteosomal degradation, becomes accumulated in the cytosol and traslocates to the nucleus activating the expression of Wnt target genes (Gordon and Nusse, 2006; Kim et al., 2009). In addition to its role in Wnt signaling, β-catenin plays crucial roles in cell–cell contacts (Nelson and Nusse, 2004). In turn, BMP ligands bind to pre-formed heteromeric complexes of BMP receptors type I and type II (BMPRI and BMPRII) to induce the phosphorylation of the cytosolic Smad proteins that migrate to the nucleus to induce target genes (Nohe et al., 2002; Shi and Massague, 2003; Miyazono et al., 2005). In addition, BMPs also have the ability to trigger Smad-independent pathways (Sieber et al., 2009; Bragdon et al., 2011). Signaling through Wnt and BMP ligands exert a wide range of effects that are highly dependent on the cell type; therefore, indentifying critical factors such as co-receptors, antagonists and intracellular regulatory proteins, is required to understand the potential contribution of these signaling pathways in different physiological contexts.

Wnt pathways are not only involved in the formation of neuronal connectivity, but also in synaptic function and plasticity (Cerpa et al., 2011; Salinas, 2012). Remarkably, several lines of research support the idea that a decrease in Wnt signaling activity is associated to the pathogenesis of some neurodegenerative diseases, such as Alzheimer’s and Parkinson’s (Inestrosa and Arenas, 2010; L’Episcopo et al., 2011). Consistent with this notion, activation of Wnt signaling plays neuroprotective roles in models of Alzheimer’s disease, either *in vivo* or *in vitro* (De Ferrari et al., 2003; Alvarez et al., 2004; Cerpa et al., 2010; Purro et al., 2012). In this regard, recent evidence shows that some Wnt ligands are up-regulated in motor neurons of ALS model mice (Chen et al., 2012; Li et al., 2013; Wang et al., 2013). Regarding BMP-dependent signaling, it has been demonstrated that the BMP2 ligand is up-regulated in damaged motor axons of the facial nerve, suggesting that changes in the activity of BMP pathways could be involved in protection or regeneration of motor neurons (Wang et al., 2007; Henriquez et al., 2011).

In this work, we first characterized motor neuron-like NSC34 cells stably expressing wild-type or G93A mutated forms of human SOD1 (Gomes et al., 2008). ALS-like cells displayed Golgi fragmentation, as well as impaired morphological differentiation and lower expression levels of the motoneuron marker Hb9 than control cells. Also, cell death was significantly higher in differentiated cells expressing mutant hSOD1. Regarding signaling, Wnt-dependent transcription was inhibited in these cells, a finding likely associated to an altered distribution of β-catenin. In turn, the BMP/Smad-dependent pathway was increased in ALS-like cells. Our findings suggest that Wnt and BMP-dependent pathways could play relevant functions in the context of motor neuronal cell death occurring in ALS.

## MATERIALS AND METHODS

### CELL CULTURE

Neuroblastoma × spinal cord cells NSC34 (Cashman et al., 1992) stably expressing human wild-type SOD1 (NSC34hSOD1WT cells) or mutant SOD1 (NSC34hSOD1G93A cells) were a gently gift of Dr. Julia Costa at ITQB, Oerias, Portugal (Gomes et al., 2008). Cells were grown in Dulbecco’s modified Eagle’s medium (DMEM; Hy-Clone, South Logan, UT, USA) supplemented with 15% fetal bovine serum (FBS) 1% penicillin/streptomycin solution and 0.4 mg/ml G418 at 37°C in a 5% CO2 atmosphere. Cells were grown on plastic or glass surfaces previously coated with 0.01% poli-L-lysine (Sigma Aldrich, Saint Louis, MO, USA) for 24 h at 37°C, and 0.5% gelatin (Sigma) for 30 min at 37°C. Cells were induced to differentiate in Neurobasal medium (Invitrogen, Grand Island, NY, USA) without FBS for 24–36 h.

### REVERSE TRANSCRIPTION-POLYMERASE CHAIN REACTION

Total RNA from NSC34 cells was obtained using Trizol reagent (Invitrogen). For reverse transcription-polymerase chain reaction (RT-PCR), 1 μg of RNA was pretreated with DNase I (Fermentas, ON, Canada) and further incubated in a buffer containing 10 μM oligo dT, reverse transcription buffer (0.5 M Tris–HCl, pH 8.3, 0.75 M KCl, 0.03 M MgCl2), 20 U RNase inhibitor (NEB, Ipswich, MA, USA) and 1 mM dNTPs (Invitrogen) at 37°C for 5 min. Stratascript reverse transcriptase (Stratagene, Baltimore, MD, USA) was added (160 U) and the mix was further incubated at 42°C for 1 h. Parallel reactions were performed in the absence of reverse transcriptase to control for the presence of contaminant DNA. For amplification, a cDNA aliquot in a volume of 12.5 μl containing 20 mM Tris buffer pH 8.4, 50 mM KCl, 1.6 mM MgCl2, 0.4 mM dNTPs, and 0.04 U Taq polymerase (Kapabiosystems, Boston, MA, US) was incubated 95°C for 5 min, 95°C for 30 s, 50°C for 30 s, and 72°C for 30 s for 35 cycles. Primers were Hb9_S: GTACCTGTCTCGACCCAAGC, Hb9_AS: CCATTGCTGTACGGGAAGTT (expected product 327 bp), GAPDH_S: GGAGCCAAACGGGTCATCATCTC, GAPDH_AS: GAGGGGCCATCCACAGTCTTCT (expected product 233 bp) BMPRII_S: TTTGCAGCCTGTGTGAAGTC, BMPRII_AS: CACAAGCTCGAATCCCTAGC (expected product 403 bp). PCR products were separated by 1.2% agarose gel electrophoresis and visualized following ethidium bromide staining.

### WESTERN BLOT

Cells were lysed in Tris-HCl 50 mM, pH 7.5; NaCl 100 mM, Triton X-100 0.5 % v/v buffer. Equal amounts of protein were resolved on SDS-polyacrylamide gels, transferred onto PVDF membranes (Millipore, Billerica, MA, USA) and subjected to Western blot analyses. Antibodies against α-tubulin (Sigma-Aldrich, St. Louis, MO, USA), Id1 (Santa Cruz Biotechnology, Santa Cruz, CA, USA), β-Catenin (Santa Cruz), phospho-Smad 1/5/8 (pSmad) (Cell Signaling Technologies, Frankfurt, Germany), and Hb9 (Abcam, Cambridge, UK) were used for immunoblotting. Bound antibodies were visualized using horseradish peroxidase-coupled secondary antibodies (Jackson InmunoResearch, West Grove, PA, USA) followed by development under a chemiluminescence kit (Perkin Elmer, Waltham, MA, USA).

### IMMUNOFLUORESCENCE MICROSCOPY

For immunostaining, NSC34 cells were grown on 18 mm glass coverslips. When indicated, cells were treated with 40 mM lithium chloride for 6 h. The medium was removed and cells were rinsed with cold PBS, fixed with 4% paraformaldehyde for 30 min, or acetone/methanol (1:1) for 5 min, at 4°C and subsequently permeabilized with 0.1% Triton X-100 in Tris-phosphate buffer. Cells were rinsed with Tris-phosphate and then incubated with primary antibodies diluted in blocking solution (1% BSA in Tris-phosphate buffer), 15 h at 4°C. Primary antibodies were anti-Islet1 (1:10; Developmental Studies Hybridoma Bank); anti-MAP1B (1:450; Santa Cruz); anti-β-Catenin (1:200; Santa Cruz), and anti Hb9 (1:100; Abcam). Corresponding Alexa-488 and -546 conjugated secondary immunoglobulins (Invitrogen) were incubated for 2 h at room temperature. Images were acquired with a laser confocal LSM700 Zeiss microscope at the CMA Bio–Bio facility (Universidad de Concepción, Concepción, Chile).

### TRANSIENT TRANSFECTION

Cells were incubated in OptiMEM medium (Invitrogen) and transfected using a Lipofectamine and Plus Reagent mix (Invitrogen), according to the indications of the manufacturer. For different luciferase reporter assays, the amounts of plasmid DNAs were: pId1-luc (Lopez-Rovira et al., 2002) 1.8 μg, pRL-SV40 180 ng, and TOPFlash 0.7 μg, pRL-SV40 70 ng. To determine the morphology of the Golgi apparatus, cells were transfected with 700 ng the FU-GolgimRFP plasmid, coding for the Golgi β-galactosiltransferase protein fused to red fluorescent protein (a gently gift of Dr. P. Zamorano, Universidad de Antofagasta, Chile).

### LUCIFERASE ASSAYS

Transfected cells in 35 mm plates were split 24 h after transfection and induced to differentiate in serum free Neurobasal medium for the indicated times. Luciferase activity was quantified using the Luciferase Assay System (Promega, Madison, WI, USA). Results were normalized against a Renilla luciferase control reporter vector (Promega).

### CELL VIABILITY ASSAY

Cells grown in 35 mm plates were treated with 0.2 mM hydrogen peroxide for 30 min at 37°C. After washing, cells were incubated with growing or differentiation medium and returned to the incubator for additional 6 h. Cell viability was measured by the LDH release assay based on the instructions of the manufacturer (VALTEK, Santiago, Chile). LDH activity was quantified in conditioned media and in cell extracts obtained in buffer Tris-HCl 50 mM, pH 7.5, NaCl 100 mM, Triton X-100 0.5% v/v, at the indicated times.

### ADHESION ASSAY

Cell culture substrates were prepared by coating Petri plates with nitrocellulose by dissolving 5 cm^2^ of nitrocellulose in 6 ml of methanol (Lagenaur and Lemmon, 1987). Droplets of 2 μl containing substrates were applied. Substrates were 3.6 mg/ml collagen, 1% gelatin, 2 mg/ml poli-L-lysine, 1 mg/ml laminin (Sigma). After 1 h at 37°C, plates were washed and subsequently blocked with 1 % BSA. Cells were added and incubated for 2 h at 37°C. After washing, bound cells were fixed with 4% paraformaldehyde for 30 min at 4°C. Images were acquired with an Olympus CK40 microscope.

### PROLIFERATION ASSAY

Plastic 35 mm plates were seeded with 250,000 cells and trypsinized every day for three consecutive days. Cells were manually counted with a hemacytometer using an Olympus CK40 microscope. Results correspond to the average ± SEM of three independent experiments.

### IMAGE ANALYSIS

Acquired images were analyzed using the ImageJ software. The number of differentiated cells and the length of their neurites were determined in cells having at least one neurite with a minimum size equal to the cell soma diameter. For each condition, 20–25 fields from three different experiments were evaluated. The mean ± SEM intensity was plotted.

### STATISTICAL ANALYSIS

One-way ANOVA was used for comparison among three or more groups followed by Bonferroni’s *post hoc* analysis for multiple comparison between different groups. Neurite length and TOPFlash activity were compared using unpaired *t*-test. *p* < 0.05 was considered to indicate statistical significance.

## RESULTS

### CULTURE CONDITIONS FOR NSC34hSOD1 CELLS

**FIGURE 1:**
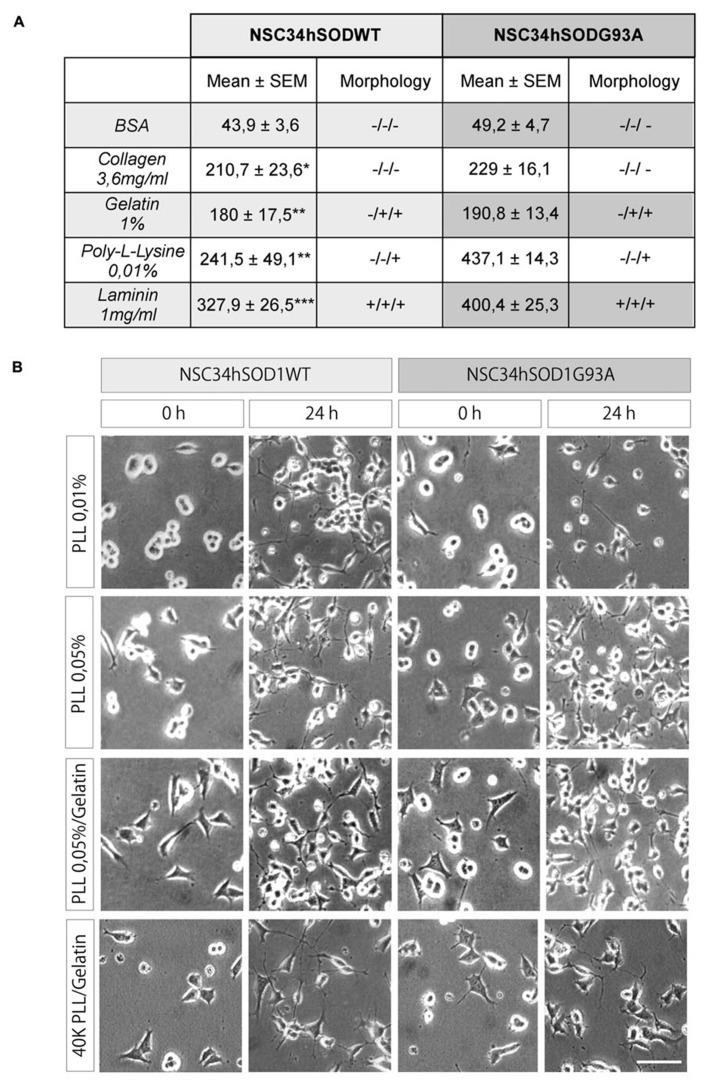
**PLL/gelatin coating is a good adhesion and differentiation substrate for NSC-hSOD1 cells.** NSC34hSOD1 cells bind to different adhesion substrates. **(A)** Culture dishes were coated with methanol-solubilized nitrocellulose. Droplets containing collagen, gelatin, PLL, BSA, and laminin were applied to the surface of the dishes and dried. NSC34hSOD1 cells were then incubated for 2 h and examined by phase contrast microscopy. Table represents the average ± SEM of three independent experiments (^*^p < 0.05, ^*^^*^p < 0.001, ^*^^*^^*^p < 0.001, ANOVA) as well as a morphological evaluation of the adhesion. **(B)** Culture dishes were coated with the indicated concentrations of PLL for 24 h. When indicated, gelatin was added for 30 min. Cells were allowed to adhere for 36 h, and differentiated for additional 24 h and analyzed by phase contrast microscopy. A total of 60,000 cells were seeded in every condition, except for the lowest panel pictures, where the number was diminished to 40,000 cells. Bar, 50 μm.

As NSC34 cells stably expressing human SOD1, either the wild-type or the G93A mutated form, adhere poorly to plastic or glass culture surfaces, we first searched for a homogeneous and reproducible surface coating that allows good cell adhesion and differentiation. We begun evaluating commonly used coating substrates for cell adhesion such as gelatin, collagen, and poly-L-lysine, using bovine serum albumin and laminin as negative and positive controls, respectively. Quantification of the data shows that poly-L-lysine and gelatin were the best substrate of the series, as they allowed the adhesion of significantly higher amounts of cells than the control BSA (see table in **Figure 1A**). As we observed that cells seeded on poly-L-lysine showed a round and refringent aspect, whereas cells on gelatin assumed a flattened polygonal morphology (see table in **Figure 1A**), we tested mixtures of substrates. **Figure 1** (two lower panels) shows that cells seeded on a surface containing poly-L-lysine plus gelatin adhered in suitable number and morphology, as compared to cells plated onto poly-L-lysine alone (**Figure 1**, two upper panels). In addition, we found that cells seeded on poly-L-lysine plus gelatin also displayed better morphological differentiation than cells plated on poly-L-lysine alone, characterized by cells extending processes which origin and end are clearly distinguishable (**Figure 1**, 24 h). This morphological feature was better observed after decreasing the amount of plated cells (**Figure 1**, lowest panel). In summary, we setup optimal conditions for the amplification, adhesion, and differentiation of NSC34hSOD1 cells.

### NSC34hSODG93A CELLS AS A MODEL OF ALS MOTOR NEURONS

**FIGURE 2:**
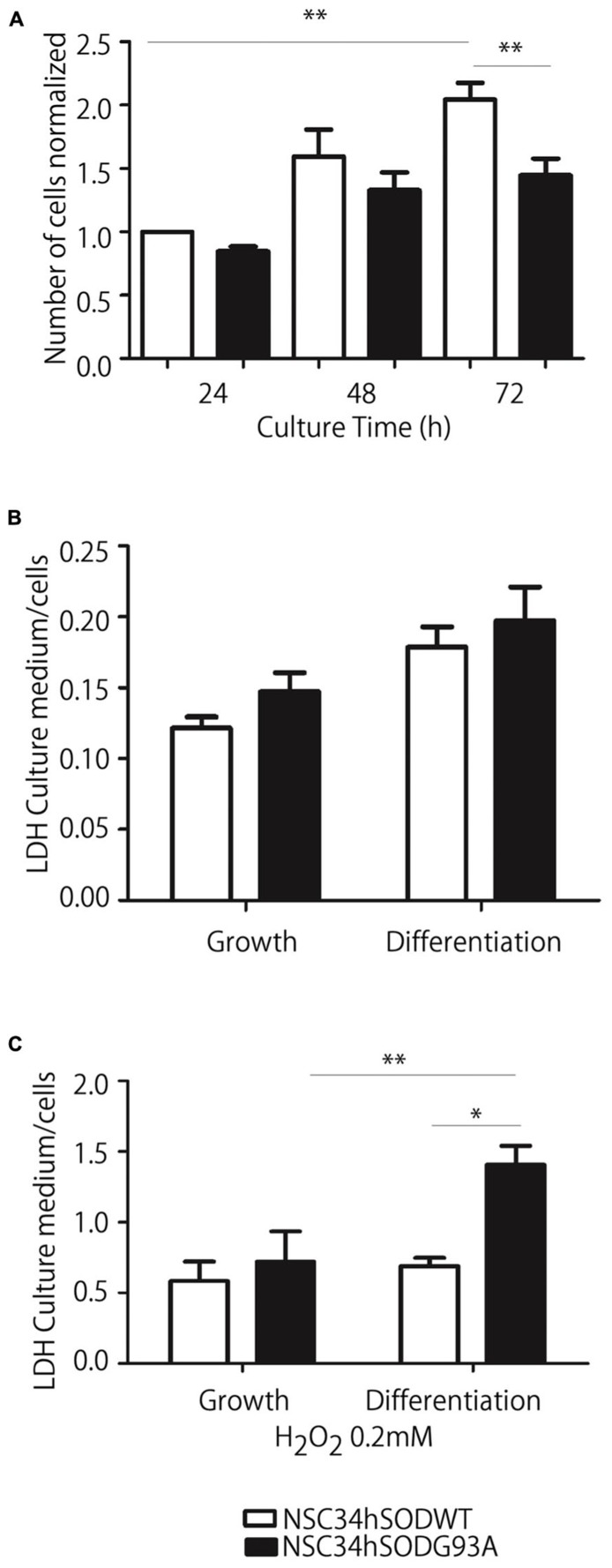
**NSC34-hSOD1G93A cells proliferate less efficiently and are more susceptible to oxidation-induced cell death. (A)** Cells were seeded in p35 plastic dishes and kept in growing medium for up to 72 h. Cells were tripsinized every 24 h and counted. Plot represents the average ± SEM of three independent experiments performed by triplicate (^*^^*^p < 0.001, ANOVA). **(B,C)** NSC34-hSOD1 cells were seeded in PLL/gelatin-coated plastic dishes and differentiated **(B)** or treated with H2O2 and differentiated **(C)**. Plots represent the ratio of LDH concentration in conditioned media against that in cell extracts, and is the average ± SEM of three experiments (^*^p < 0.05, ANOVA).

We next conducted a series of experiments aimed to assess the reliability of NSC34hSOD1 cells to be used as a model of ALS motor neurons. First, we evaluated the effect of the stable expression of hSOD1 on cell proliferation by counting the number of cells seeded onto uncoated plastic dishes every 24 h. While both NSC34hSOD1 cell lines grow 0.5-fold during the first 48 h, only NSC34hSOD1WT cells continued proliferating at 72 h (2.01 ± 0.1 times, compared to 24 h; ^*^^*^p < 0.05). Indeed, the number of NSC34hSODG93A cells remained constant and was significantly lower than NSC34hSOD1WT cells at the same time point (**Figure 2A**), revealing that the stable expression of hSODG93A results in an impaired proliferation rate of NSC34 cells.

Second, considering that ALS cells are more sensitive to oxidative stress than controls (Wijesekera and Leigh, 2009), we evaluated the survival of NSC34hSOD1 cells in basal and oxidative conditions (0.2 mM hydrogen peroxide) by quantifying LDH activity in conditioned media and extracts of both cell lines. In basal conditions, cell survival was not different in growing and differentiated conditions of both cell lines (**Figure 2B**). However, exposure to an oxidative condition induces a strong increase in LDH release of differentiated NSC34hSODG93A cells compared to those cultured in growing conditions; indeed, extracellular LDH is significantly higher in differentiated NSC34hSODG93A than control NSC34hSODWT cells (**Figure 2C**). Thus, NSC34 cells expressing hSODG93A are more susceptible to oxidation-induced cell death.

**FIGURE 3:**
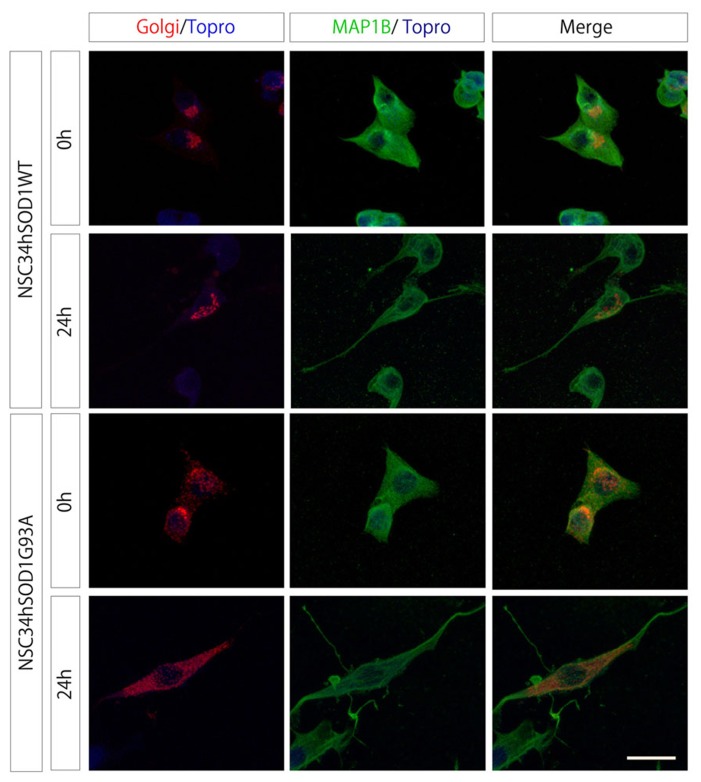
**NSC34hSOD1G93A cells display Golgi disruption.** NSC34hSOD1 cells were transfected with the FU-Golgi-mRFP vector, and fixed at 0 and 24 h of differentiation to determine the subcellular localization of Golgi fragments. Immunocytochemistry against MAP1B was used to stain the cell cytoplasm and nuclei counter staining Topro-3. Bar, 20 μm.

Third, we considered that ALS motor neurons display fragmentation of their Golgi apparatus (Mourelatos et al., 1994; Turner et al., 2005; Gomes et al., 2008; Sundaramoorthy et al., 2013). In order to determine the temporal appearance of Golgi disruptions, cells were transfected with a plasmid coding for a β-galactosyltransferase fused to mRFP and subsequently differentiated. In NSC34hSOD1WT cells, the Golgi staining distributes, as expected, in the periphery of the nucleus, either in undifferentiated cells as well as in those differentiated for 24 h (**Figure 3**). Remarkably, even though the labeling also distributes in the perinuclear region of undifferentiated NSC34hSOD1G93A cells, Golgi portions are also disseminated within cytoplasmic regions (**Figure 3**). In differentiated NSC34hSOD1G93A cells, Golgi fragments were not concentrated in any particular subcellular region and appear regularly distributed within the cytoplasm (**Figure 3**). Taken together, our results show NSC34hSOD1 cells phenocopy what has been observed in patients as well as in validated models of ALS. Also, they suggest that Golgi disruption could be an early event of ALS pathogenesis.

**FIGURE 4:**
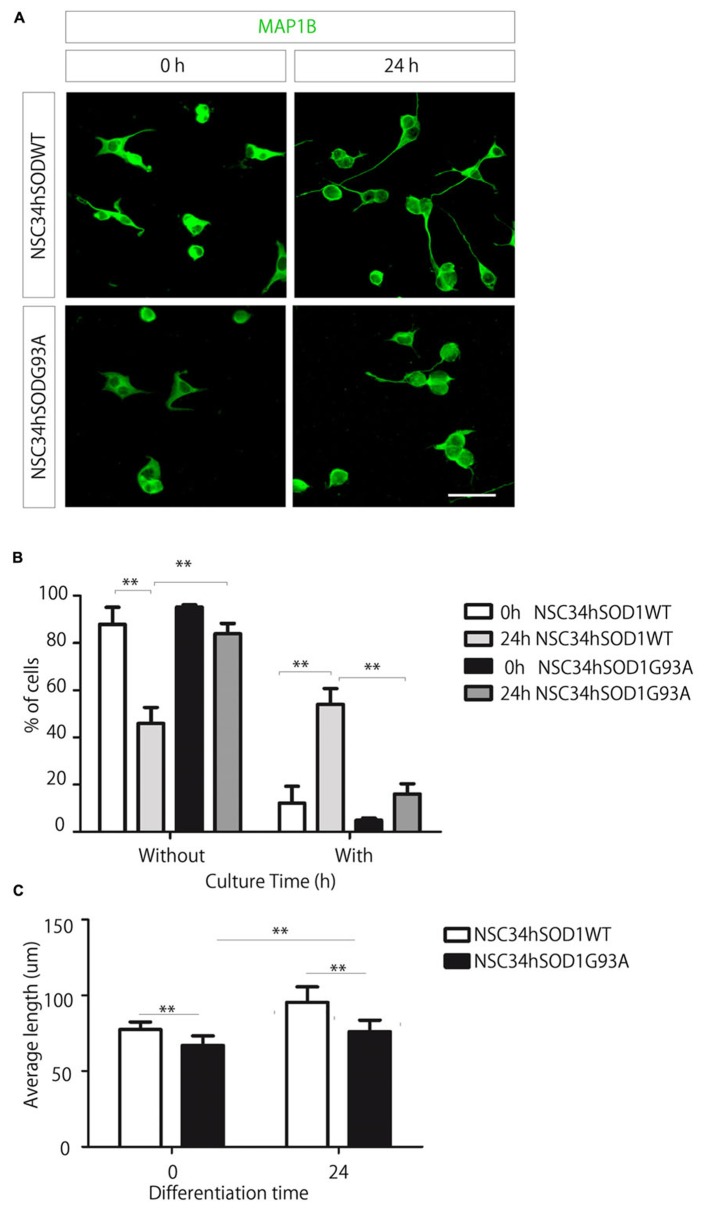
**A small proportion of NSC-hSOD1G93A cells display neurite outgrowth.** NSC34hSOD1WT and NSC34hSOD1G93A cells were plated in PLL/gelatin-coated coverslips and differentiated for 24 h. Cells were fixed and stained against MAP1B **(A)**. Different parameters of morphological differentiation, including percentage of cells with or without neurites **(B)** and average neurite length **(C)** were measured using ImageJ. Plots are the average ± SEM of three independent experiments (^*^^*^p < 0.01, ANOVA; ^*^p < 0.05, *t* test). Bar, 50 μm.

Fourth, we evaluated the ability of NSC34hSOD1 cells to differentiate into a neuronal-like phenotype (**Figure 4**). Cells were seeded onto coated glass coverslips, differentiated for 24 h and subsequently stained against MAP1B (Benavente et al., 2012). Even though both cell lines extend neurites after 24 h, this feature is impaired in NSC34hSODG93A cells (see **Figures 1** and **4A**, lowest panel). Indeed, quantification of the data reveals an expected significant increase of NSC34hSOD1WT cells bearing neurites upon differentiation (from 12.1 ± 7.1 to 54.0 ± 6.7%; ^*^^*^p < 0.01), while the proportion of NSC34hSOD1G93A cells having neurites was not significantly higher than undifferentiated cells (**Figure 4B**). Similarly, the average length of the neurites was higher in control NSC34hSOD1WT than in NSC34hSOD1G93A cells, either before (77.4 ± 4.9 vs. 66.8 ± 6.4 μm; ^*^p < 0.05) and after differentiation (95.3 ± 10.3 vs. 76.6 ± 7.5 μm; ^*^p < 0.05; **Figure 4C**). These results suggest that the constitutive expression of hSOD1G93A impairs the ability of NSC34 cells to differentiate.

**FIGURE 5:**
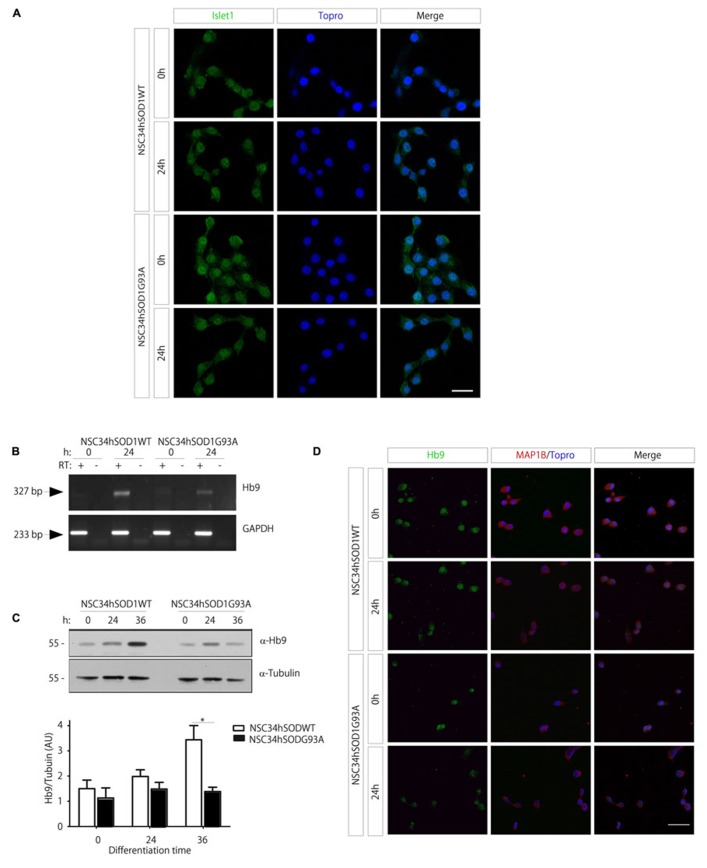
**Expression of motor neuron cell markers in NSC34hSOD1 cells. (A)** NSC34hSOD1 cells were seeded onto glass coverslips and differentiated for 24 h. Immunocytochemical analyses show that Islet-1 is localized at the cell nuclei, which were counterstained with ToPro-3. **(B)** Total RNA was extracted at 0 and 24 h of NSC34hSOD1 cell differentiation, and subsequently subjected to reverse transcription (RT^+^) to obtain cDNA. As a control, the same reaction was performed in the absence of reverse transcriptase (RT^-^). PCRs were performed with specific primers to amplify Hb9 and the internal control GAPDH. **(C)** Western blot analyses were performed with specific antibodies against Hb9 and the internal control α-tubulin. The bottom panel shows quantification of the data, which is expressed as the mean ± SEM of four independent experiments (*p < 0.05). **(D)** Immunocytochemical analyses show that Hb9 is localized in the nuclei of all cells, but the intensity of the staining is less evident in differentiated NSC34hSOD1G93A cells. Cells were also stained with MAP1B and nuclei with ToPro-3. Bar, 20 **(A)** and 50 μm **(D)**.

Our findings thus far show that several features of the physiopathology of ALS are evident at particular time points of the differentiation of NSC34hSOD1 cells. Even though NSC34 cells have been extensively used as a valid model of motor neurons (Cashman et al., 1992), their molecular identity has not been fully described. In this context, we first analyzed the expression of Islet-1, a transcription factor early expressed and necessary for the differentiation of motor neurons (Ericson et al., 1992; Pfaff et al., 1996). Immunocytochemical analyses showed that Islet-1 is constitutively expressed in both cell lines, in growing and differentiated conditions, and becomes mainly concentrated in the cell nuclei (**Figure 5A**), suggesting that the early commitment of NSC34 cells to the motor neuron phenotype is not affected by the stable expression of hSOD1. We then followed the expression of Hb9, a transcription factor selectively expressed by developing motor neurons (Pfaff et al., 1996). Hb9 expression suffices to induce the differentiation of post-mitotic cells, whereas its absence results in misslocalized motor axons in distal muscle regions (Arber et al., 1999). PCR analyses showed that the expression of Hb9 mRNA is increased at 24 h of the differentiation of NSC34hSOD1WT cells, but not in NSC34hSOD1G93A cells (**Figure 5B**). These results were corroborated at the protein level following Western blot analyses (**Figure 5C**). Quantification of the data reveals that Hb9 expression is significantly higher in differentiated NSC34hSOD1WT, compared to NSC34hSOD1G93A cells (**Figure 5C**, lower panel). Consistent with these findings, our immunocytochemical analyses showed that, even though all cells distribute Hb9 in the nucleus, the intensity of the staining is reduced in differentiated NSC34hSOD1G93A cells (**Figure 5D**). These findings suggest that an ALS condition could interfere with the ability of neuronal precursors to acquire the motor neuronal fate during early embryonic development.

### Wnt- AND BMP-DEPENDENT SIGNALING IN NSC34hSOD1WT AND NSC34hSODG93A CELLS

Disfunction of Wnt signaling has been associated to neurodegenerative conditions such as Alzheimer’s and Parkinson’s disease (Inestrosa and Arenas, 2010; L’Episcopo et al., 2011; Marchetti et al., 2013). More recently, the expression levels of mRNAs coding for proteins related to Wnt signaling have also been proven to be altered in mouse models of ALS (Yu et al., 2013).

**FIGURE 6:**
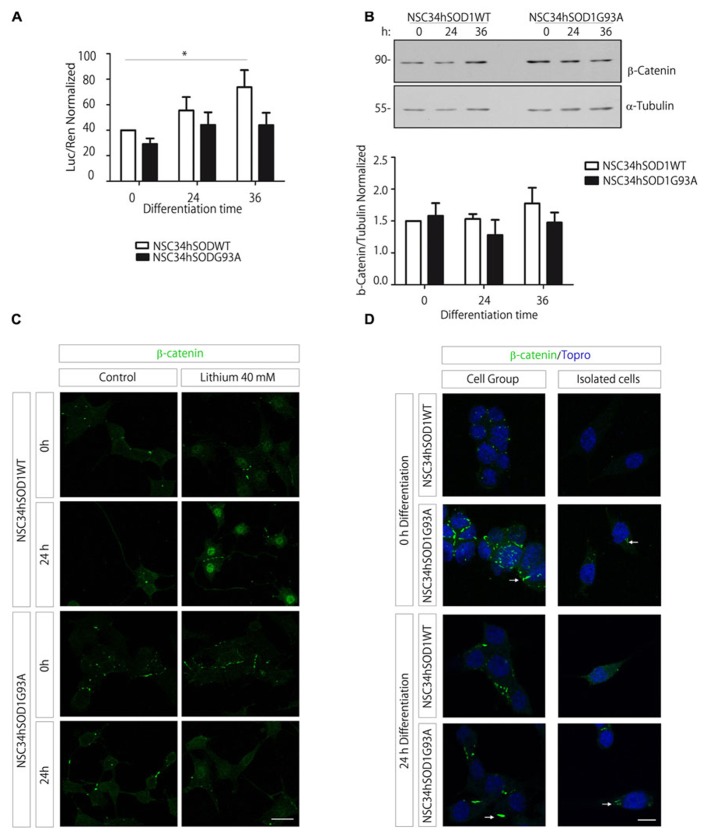
**The Wnt/β-catenin signaling increases in control NSC34hSOD1WT cells but not in ALS-like NSC34hSOD1G93A cells, possibly due to the presence of β-catenin aggregates. (A)** Cells were seeded on PLL/gelatin-coated dishes and transfected with the Wnt reporter TOPFLASH vector plus the pRLSV40 Renilla control plasmid, and subsequently differentiated for the indicated times. The graph shows the normalized reporter gene activity (luciferase/Renilla). Data are expressed as the average ± SEM of three independent experiments performed by quadruplicate (^*^p < 0.05, paired *t* test). **(B) **Total protein extracts were obtained from differentiating NSC34hSOD1 cells at 0, 24, and 36 h and were subjected to western blot analyses using an anti β-catenin antibody. An anti α-tubulin antibody was used as loading control. The graph shows the quantification of β-catenin normalized to α-tubulin bands, and represent the average ± SEM of three independent experiments. **(C)** Undifferentiated and differentiated NSC34hSOD1 cells were treated with 40 mM lithium chloride for 6 h and stained with an anti β-catenin antibody. Lithium treatment increases the nuclear staining of β-catenin in control cells, a feature that is not evident in ALS-like cells. **(D)** NSC34hSOD1 cells were fixed before and after 24 h of differentiation and subsequently subjected to immunocytochemical analyses using an anti β-catenin antibody. Grouped and isolated cells are shown for each condition. Nuclei were counterstained with ToPro-3. Bar, 20 **(C)** and 10 μm **(D)**.

We first analyzed the transcriptional activation of the Wnt/β-catenin pathway following the activity of a Wnt reporter gene. Our findings show a significant increase during the differentiation of control NSC34hSOD1WT cells, whereas such activation was not observed in NSC34 cells expressing the G93A mutation of hSOD1 (**Figure 6A**). Based on these findings, we next studied the expression profile of the key Wnt mediator β-catenin. Western blot analyses showed that the total amount of β-catenin was not significantly modified during the differentiation of both NSC34hSOD1 cell lines (**Figure 6B**). Next, we analyzed the distribution of β-catenin following activation of the pathway with lithium chloride (Alvarez et al., 2004). The intensity of nuclear β-catenin staining becomes strongly increased in the control cell line upon lithium treatment, being more evident in differentiated cells (**Figure 6C**). In turn, the localization of β-catenin in the nuclei of ALS-like cells was virtually absent in undifferentiated cells and very faint in differentiated ones (**Figure 6C**). Nevertheless, the distribution of β-catenin in the cytoplasm displayed pronounced modifications. In control cells most β-catenin is found in focal points of cell–cell contacts, either in differentiation and growing conditions. In turn, these structures are more abundant and larger in NSC34hSOD1G93A cells, a feature that is particularly evident in undifferentiated cells (**Figures 6C,D**). In isolated cells, aggregates of cytosolic β-catenin were only detected in NSC34hSOD1G93A cells (arrows in **Figure 6D**). Together, these findings suggest that canonical Wnt signaling is impaired in ALS-like cells, possibly due to the altered distribution of the key effector β-catenin.

**FIGURE 7:**
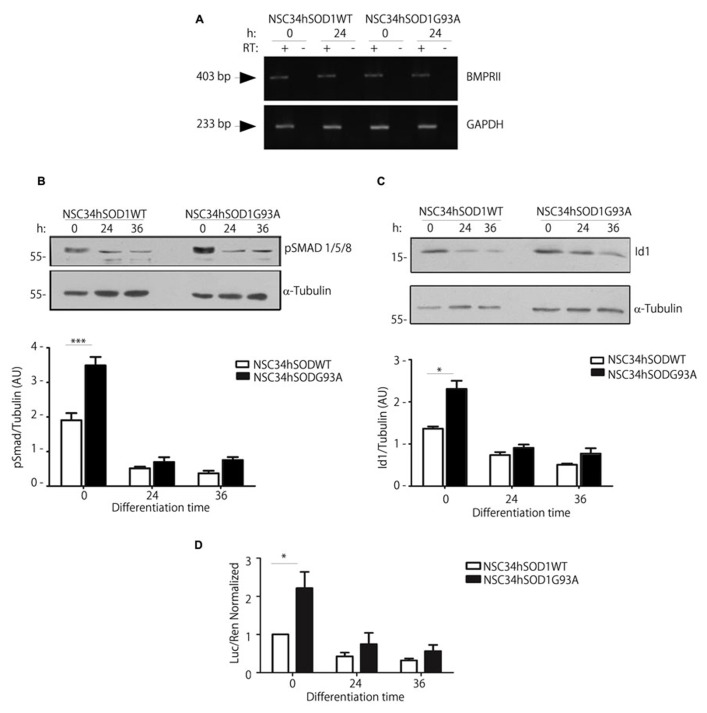
**Undifferentiated NSC34hSOD1G93A cells display increased activity of the BMP/Smad signaling pathway. (A)** Total RNA was obtained from NSC34hSOD1 cells at 0 and 24 h of differentiation and BMPRII mRNA levels were analyzed by RT-PCR. As loading control GAPDH was amplified. Negative control reactions without reverse transcriptase (RT^-^) are also shown. **(B,C)** Western blot analysis using anti pSmad **(B)** or anti Id1 **(C)** antibodies in protein extracts of NSC34hSOD1 cells obtained at the indicated times of differentiation. Anti α-tubulin was used as loading control. Data analyses of band intensities are expressed as the mean ± SEM of three independent experiments (^*^p < 0.05; ^*^^*^^*^p < 0.01). **(D)** NSC-34 cells were transfected with an Id1 promoter luciferase (Luc) reporter gene. Enzyme activity was measured before or after 24 and 36 h of differentiation and normalized by Renilla (Ren) luciferase activity. Data are the mean ± SD of three independent experiments performed in quadruplicate (^*^p < 0.05).

Our recent findings show that BMP-dependent pathways could play relevant roles on the differentiation of motor neurons (Benavente et al., 2012). As a first hint to analyze BMP signaling in the context of ALS, we studied the expression of the mRNA coding for the type II BMP receptor (BMPRII), which signals through either Smad and non-Smad pathways (Sieber et al., 2009; Henriquez et al., 2011). Our results show that BMPRII expression was not modified during the differentiation of both NSC34hSOD1 cells, as compared to the internal control GAPDH (**Figure 7A**). Next, we performed western blot analyses to detect the phosphorylation of Smad1/5/8, a crucial step in the activation of the Smad-dependent BMP pathway (Henriquez et al., 2011). Similar to our previous findings in parental NSC34 cells (Benavente et al., 2012), pSmad is down-regulated during the differentiation of both NSC34hSOD1 cells; however, pSmad basal levels are significantly higher in undifferentiated NSC34hSODG93A cells, compared to controls (**Figure 7B**). Then, in order to correlate pSmad levels with BMP-dependent transcription, we analyzed the expression and activity of the Smad-dependent early responsive gene Id1, a negative regulator of the differentiation of neurogenic precursors (Ying et al., 2003; Vinals et al., 2004). Immunodetection experiments showed that Id1 levels in undifferentiated cells were significantly higher in NSC34hSOD1G93A cells compared to controls, whereas its expression is similarly down-regulated during the differentiation of both cell types (**Figure 7C**). Finally, we transiently transfected undifferentiated NSC34hSOD1 cells with a luciferase Smad-responsive Id1 reporter gene (Lopez-Rovira et al., 2002). Consistent to our findings on pSmad and Id1 expression levels, **Figure 7D** shows that the basal Id1-dependent transcription is twofold higher in NSC34hSOD1G93A cells compared to control NSC34hSOD1WT cells, whereas this activity is strongly diminished after 24 and 48 h of differentiation in both cell types. Together, these findings support the notion that BMP/Smad signaling varies inversely with motor neuron differentiation; in addition, they also suggest that this signaling pathway could be up-regulated in ALS motor neuron precursor cells.

## DISCUSSION

Amyotrophic lateral sclerosis is a neurodegenerative disease that leads to progressive paralysis and death after around 4 years from the onset of the disease. Since its approval, the only treatment for ALS is Riluzole, a drug that inhibits the release of glutamate by inactivation of voltage-sensitive sodium channels (van Zundert et al., 2012), extending 3 months on average. Therefore, it is critical to generate model systems to describe the physiopathology of the disease and where to assess more effective therapeutic alternatives.

Because of its origin, NSC34 cells exhibit several features of motor neurons (Cashman et al., 1992), and are a more suitable *in vitro* model to study ALS than other neuronal cell lines that have also been used for similar analyses such as the Neuro-2a and SH-SY5Y cells (Pasinelli et al., 1998; Arciello et al., 2011). In their initial characterization, both NSC34hSOD1 cell types adhered well to Matrigel/poly-D-lysine matrices (Gomes et al., 2008). As Matrigel contains most of the components of the extracellular matrix, likely influencing cell behavior (Hughes et al., 2010; Ventre et al., 2012), we looked for simpler coating molecules. We found that that the poly-L-lysine plus gelatin mixture allowed good cell adhesion and differentiation of NSC34hSOD1 cells. In agreement with our results, both poly-L-lysine and gelatin have been extensively used by their ability to significantly increase cell adhesion in various cell types while not affecting cell proliferation or differentiation (Alvarez Perez et al., 2012; Kuo and Chung, 2012).

Several molecular mechanisms are likely to converge to allow motor neuronal degeneration, the main feature of ALS (Pasinelli and Brown, 2006; Wijesekera and Leigh, 2009; Gordon and Meininger, 2011; Bendotti et al., 2012). In this regard, our results revealing that NSC34hSOD1G93A cells are more prone to oxidation-induced cell death are supported by previous findings showing that NSC34 cells expressing hSOD1G93A increase reactive oxygen species production, while decreasing their viability (Li et al., 2003). In addition, other detrimental stimuli, such as staurosporine treatment, induced the death of NSC34hSOD1G93A cells (Gomes et al., 2008), reinforcing the notion that the expression of mutant SOD1 increases the vulnerability of motor neurons to cell damage.

Another hallmark of ALS neuropathology is the fragmentation of their Golgi apparatus, a feature that has been observed in ALS patients (Mourelatos et al., 1994), as well as in various model systems, such as NSC34 cells expressing or treated with hSOD1G93A (Mourelatos et al., 1994; Gomes et al., 2008), and transgenic mice expressing hSOD1G93A (Turner et al., 2005). Interestingly, our present findings reveal that Golgi fragmentation occurs prior to neuronal differentiation. In this regard, it has been shown that mutated hSOD1 is released from NSC34hSOD1G93A cells, as well as from primary astrocyte cultures derived from hSOD1G93A transgenic mice, via exosomes (Gomes et al., 2007; Basso et al., 2013). These vesicles are then endocytosed by neuronal cells inducing Golgi fragmentation (Sundaramoorthy et al., 2013). Considering these previous findings supporting the notion that ALS-inducing stimuli could be spread between cells, our present results strongly suggest that such mechanism could take place from early steps of the pathogenesis of the disease.

Consistent with previous findings showing that NSC34 cells expressing hSOD1G93A display impaired morphological differentiation (Gomes et al., 2008; Magrane et al., 2009), and decreased expression of the neuronal marker MAP2 (Lee et al., 2002), our data show that these cells have a lower percentage of differentiated cells that bear shorter neurites than controls. What is the molecular mechanism underlying this altered phenotype? Even though NSC34 cells were obtained from neuronal precursors from the spinal cord, and have been validated as a cholinergic system (Cashman et al., 1992; Maier et al., 2013), their identity as motor neurons has not been evaluated in detail. Islet-1 is considered an early marker of differentiation of motor neurons and their absence impairs the ability of neuronal precursors to differentiate into motor neurons (Pfaff et al., 1996). In turn, Hb9 is a transcription factor expressed in post-mitotic motor neurons, which suggest that it functions later in the differentiation process (Pfaff et al., 1996; Tanabe et al., 1998). Our findings show that control cells display a constitutive expression of Islet-1 and induce the expression of Hb9 upon differentiation; however, whereas Islet-1 levels are similar in both cell lines, the expression of Hb9 is significantly impaired in ALS-like cells, indicating that the consolidation of motor neuronal identity might be compromised. In this regard, recent evidence show that motor neuron-like cells driven from iPS cells obtained from hSOD1G93A mice express several motor neuron markers, such as choline acetyl transferase and Hb9, and exhibit shorter neuronal projections than cells derived from control mice (Yao et al., 2013).

Several lines of research support the idea that a decrease in Wnt signaling activity is associated to the pathogenesis of some neurodegenerative diseases, such as Alzheimer’s and Parkinson’s (Inestrosa and Arenas, 2010; L’Episcopo et al., 2011). When hippocampal neurons are treated with the Aβ peptide (which extracellular accumulation is a hallmark of Alzheimer’s disease), there is an increase in cell apoptosis as well as in GSK-3β activity, whereas the intracellular levels of β-catenin and the transcription of Wnt-target genes are reduced (Garrido et al., 2002; De Ferrari et al., 2003; Alvarez et al., 2004). Consistent with this notion, activation of Wnt signaling plays neuroprotective roles in models of Alzheimer’s disease either *in vivo* or *in vitro* (De Ferrari et al., 2003; Alvarez et al., 2004; Cerpa et al., 2010; Purro et al., 2012). Indeed, the treatment of hippocampal slices with antibodies against the Wnt inhibitor DKK1 reverts the adverse effects observed in AD brains (De Ferrari et al., 2003; Alvarez et al., 2004; Cerpa et al., 2010; Purro et al., 2012). Together, these findings provide a strong support to a crucial role for Wnt signaling on neurodegeneration.

Recent findings show that the motor neurons of ALS model mice display altered expression of some Wnt effectors, such as Wnt1, Wnt2, Wnt5a, Fz1, and Fz2 (Chen et al., 2012; Li et al., 2013; Wang et al., 2013). Consistently, following a systematic microarray approach, Yu et al. (2013) have shown that many Wnt effectors, including Wnt ligands, Fz receptors, endogenous antagonists, downstream effectors, and target genes modify their expression in the spinal cord of ALS mice. Remarkably, when we analyzed Wnt signaling, we found that the ALS-like motor neurons did not activate the β-catenin-dependent Wnt pathway during their differentiation, as we observed in control cells.

Neurodegenerative diseases are characterized by the formation of toxic protein aggregates, either because they block cell functions or because they sequester other proteins (Wood et al., 2003; Bergemalm et al., 2010). In *in vitro* models of Alzheimer’s disease, activation of the Wnt pathway with lithium chloride results in an increase in total β-catenin levels (De Ferrari et al., 2003; Alvarez et al., 2004), as well as in increased neuronal survival (De Ferrari et al., 2003). Our present findings show that lithium treatment induced the localization of β-catenin in the nuclei of control cells, whereas this staining was significantly reduced in cells expressing mutated hSOD1. Remarkably, the intracellular distribution of β-catenin was notoriously affected, evidenced by the presence of intracellular aggregate-like structures in cell–cell contacts of NSC34hSOD1G93A cells. Interestingly, aggregates of β-catenin have been detected in *in vitro* models of AD (Ghanevati and Miller, 2005). Also, β-catenin is slightly detected in hSOD1-containing hyaline inclusions in the spinal cord of ALS model mice (Zhang et al., 2006). Therefore, it is plausible to interpret our findings as that the abnormal distribution of β-catenin alters its nuclear translocation, thus reducing transcriptional activation. In support of this view, β-catenin aggregates have been observed in the brain of patients and mice models of the neurodegenerative Huntington’s disease; moreover, the silencing of β-catenin rescued neuronal degeneration in this model system (Godin et al., 2010), suggesting that β-catenin aggregation could be crucial in the pathogenesis of ALS.

Similar to Wnt pathway, BMP signaling is also altered in the damaged adult nervous system (Wang et al., 2007; Bayat et al., 2011). We found a strong induction of Id1, Smad phosphorylation and Smad-dependent transcription in undifferentiated NSC34hSOD1G93A cells, suggesting that BMP signaling could be up-regulated during early stages of the ALS condition. Interestingly, the levels of the BMP2 ligand are up-regulated upon induction of neuronal damage (Wang et al., 2007; Matsuura et al., 2008). In this regard, we have recently demonstrated that activation of Smad-dependent signaling by treatment of NSC34 cells with the BMP2 ligand correlates with decreased morphological differentiation; however, it also induces the expression of the type II BMP receptor, which could be required for subsequent steps of regeneration (Benavente et al., 2012). In our ALS model system, the decreased morphological differentiation that correlates with up-regulation of Smad-dependent signaling was not accompanied by changes in the expression of the type II BMP receptor. Similarly, in Drosophila models of several motor diseases, including ALS, signaling mediated by the BMPRII is impaired (Ratnaparkhi et al., 2008; Hirth, 2010), thus suggesting that signaling through this receptor could also play important roles on the pathogenesis of ALS.

**FIGURE 8:**
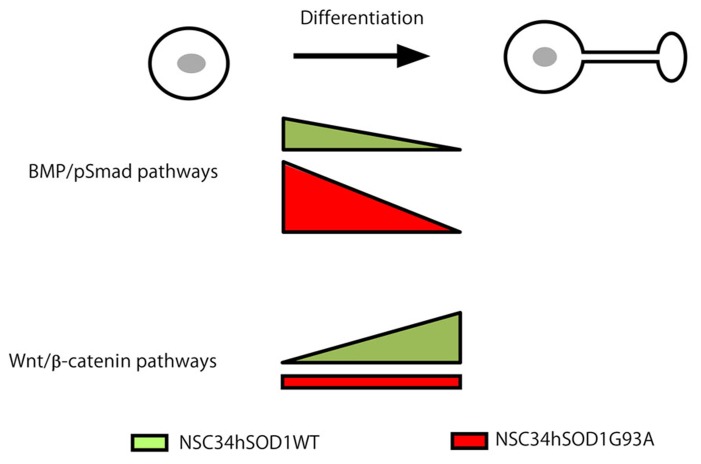
**Working model for Wnt and BMP pathways as potential mediators of ALS pathogenesis.** Wnt and BMP-dependent signaling pathways display opposite alterations at different stages of the development of ALS motor neurons. Based on our present findings we propose that BMP/Smad signaling is up-regulated at early stages of the differentiation of ALS motor neurons. In turn, the differentiated cells display an impaired ability to activate Wnt/β-catenin signaling.

Altogether, our findings fit with a model (**Figure 8**) where signaling through Wnt and BMP morphogens act as potential mediators of the pathogenesis of ALS, with opposite alterations at different stages of development; whereas BMP/Smad signaling could be up-regulated during early stages of the differentiation of ALS motor neurons, the differentiated cells could display an impaired ability to activate Wnt/β-catenin signaling. Remarkably, cumulative evidence reveals that these two signaling pathways interact in various developmental and tissue specific contexts to exert many different cellular responses (Itasaki and Hoppler, 2010; Marcellini et al., 2012). Modulating these signaling pathways individually or in a coordinated fashion could then represent interesting therapeutic alternatives to mitigate the progression of ALS.

## Conflict of Interest Statement

The authors declare that the research was conducted in the absence of any commercial or financial relationships that could be construed as a potential conflict of interest.
